# Characterization of the core and accessory genomes of *Pseudomonas aeruginosa* using bioinformatic tools Spine and AGEnt

**DOI:** 10.1186/1471-2164-15-737

**Published:** 2014-08-29

**Authors:** Egon A Ozer, Jonathan P Allen, Alan R Hauser

**Affiliations:** Department of Medicine, Division of Infectious Diseases, Northwestern University, 645 North Michigan Avenue, Suite 900, Chicago, IL 60611 USA; Department of Microbiology-Immunology, Northwestern University, 303 East Chicago Avenue, Ward 8-296, Chicago, IL 60611 USA

**Keywords:** *Pseudomonas aeruginosa*, Core genome, Accessory genome, Comparative genomics, Spine, AGEnt, Whole-genome sequencing

## Abstract

**Background:**

*Pseudomonas aeruginosa* is an important opportunistic pathogen responsible for many infections in hospitalized and immunocompromised patients. Previous reports estimated that approximately 10% of its 6.6 Mbp genome varies from strain to strain and is therefore referred to as “accessory genome”. Elements within the accessory genome of *P. aeruginosa* have been associated with differences in virulence and antibiotic resistance. As whole genome sequencing of bacterial strains becomes more widespread and cost-effective, methods to quickly and reliably identify accessory genomic elements in newly sequenced *P. aeruginosa* genomes will be needed.

**Results:**

We developed a bioinformatic method for identifying the accessory genome of *P. aeruginosa*. First, the core genome was determined based on sequence conserved among the completed genomes of twelve reference strains using Spine, a software program developed for this purpose. The core genome was 5.84 Mbp in size and contained 5,316 coding sequences. We then developed an *in silico* genome subtraction program named AGEnt to filter out core genomic sequences from *P. aeruginosa* whole genomes to identify accessory genomic sequences of these reference strains. This analysis determined that the accessory genome of *P. aeruginosa* ranged from 6.9-18.0% of the total genome, was enriched for genes associated with mobile elements, and was comprised of a majority of genes with unknown or unclear function. Using these genomes, we showed that AGEnt performed well compared to other publically available programs designed to detect accessory genomic elements. We then demonstrated the utility of the AGEnt program by applying it to the draft genomes of two previously unsequenced *P. aeruginosa* strains, PA99 and PA103.

**Conclusions:**

The *P. aeruginosa* genome is rich in accessory genetic material. The AGEnt program accurately identified the accessory genomes of newly sequenced *P. aeruginosa* strains, even when draft genomes were used. As *P. aeruginosa* genomes become available at an increasingly rapid pace, this program will be useful in cataloging the expanding accessory genome of this bacterium and in discerning correlations between phenotype and accessory genome makeup. The combination of Spine and AGEnt should be useful in defining the accessory genomes of other bacterial species as well.

**Electronic supplementary material:**

The online version of this article (doi:10.1186/1471-2164-15-737) contains supplementary material, which is available to authorized users.

## Background

*Pseudomonas aeruginosa* is a Gram-negative bacterium ubiquitously found in the environment. The organism displays a striking propensity for survival in highly diverse ecological niches and causes infections in a wide range of host organisms, including plants, nematodes, insects, and vertebrates [1]. As an opportunistic pathogen in humans, *P. aeruginosa* primarily causes infections in individuals with compromised host defenses, such as burn patients, those requiring mechanical ventilation, and patients with immune deficiencies [2–5]. The key to its survival in diverse environments as well as its broad infectious host range lies in its metabolic versatility and adaptability. For example, *P. aeruginosa* can utilize multiple carbon sources for energy, respire under aerobic and anaerobic conditions, grow at temperatures up to 42°C, form biofilms, and resist many biocides and antibiotics [6–8]. Reflective of this diversity, *P. aeruginosa* has one of the largest genomes among bacterial human pathogens, averaging 6.6 Mbp in size. As opposed to other bacterial species with expanded genomes representing gene duplication events [9], the genome of *P. aeruginosa* is made up of an assortment of genes encoding functionally diverse products. This includes a large number of genes encoding outer membrane proteins, transport systems, and enzymes involved in nutrient uptake and metabolism, as well as one of the largest proportions of regulatory genes (8.4%) among bacterial genomes [10].

The increasing availability of multiple genome sequences has made comparative genomic analyses of *P. aeruginosa* possible*.* These analyses have defined a conserved “core” genome shared among nearly all members of the species interspersed with “accessory” genomic elements that are present in some but absent in other strains of *P. aeruginosa*. Prior studies suggest that the core genome represents approximately 90% of the total *P. aeruginosa* genome and is highly conserved [11], containing the majority of genes with housekeeping functions [12]. The accessory genome of *P. aeruginosa* consists of variable-length stretches of DNA separated by core genome segments. These accessory elements are sometimes referred to as genomic islands (>10 kb) or islets (<10 kb), although the definitions of these terms are evolving to refer more specifically to genetic material with signatures suggesting horizontal transfer [13–15]. To avoid confusion, we will use the term accessory genomic element (AGE) to indicate any contiguous stretch of genetic material that is not a part of the conserved core genome, regardless of its source or structure. Major components of the *P. aeruginosa* accessory genome are integrative and conjugative elements (ICEs), replacement islands, prophages and phage-like elements, transposons, insertion sequences, and integrons [16]. Extra-chromosomal plasmids, harbored by some strains of *P. aeruginosa,* may also be considered part of the accessory genome.

The accessory genome of an individual *P. aeruginosa* strain is an important driver of its ability to persist in a particular environment. For instance, genes found in the accessory genomes of strains cultured from sites containing heavy metals and toxic organic compounds promoted persistence in these environments, which are otherwise unsuited for *P. aeruginosa* habitation [17, 18]. The bacterial accessory genome can have important clinical implications as well. In many bacteria, the accessory genome is enriched in virulence genes [19], and *P. aeruginosa* is no exception. A screen of accessory sequences in clinical strains of *P. aeruginosa* showed several open reading frames encoding proteins with homologies to bacterial virulence factors not previously seen in *P. aeruginosa* [20]. The *P. aeruginosa* genomic islands PAPI-1 and PAPI-2 encode multiple genes necessary for full virulence in animal and plant infection models [21]. For example, PAPI-2 and related islands contain the gene encoding ExoU, a type-III-secreted effector protein linked to increased virulence in animal models and human patients [22–26]. Although intrinsic antibiotic resistance mechanisms are encoded within the core genome of *P. aeruginosa*, acquired antibiotic resistance genes are present in the accessory genome [27]. The transfer of these genes between strains can contribute to outbreaks of multiply-antibiotic-resistant strains in healthcare settings [28–31].

Identification of accessory genomic elements in *P. aeruginosa* is important to the study of this organism’s evolution, niche adaptation, and infectious potential. With advances in next-generation sequencing (NGS), the sequencing of entire bacterial genomes is becoming faster, cheaper, and more accurate. For example, complete or draft sequences of 73 strains of *P. aeruginosa* had been deposited in the NCBI database at the time of this writing. Thus there is a need for a rapid method to reliably identify accessory genomic elements in newly sequenced strains of *P. aeruginosa*. Many analyses of bacterial core and accessory genomes to date have relied on annotation of query genomes and time- and resource-intensive alignment analyses to identify homologous genes among members of the species [32–34]. Several bioinformatic tools have been developed to identify genomic islands (GIs) and other non-conserved genomic elements in bacteria, but they have suffered from a variety of limitations. Comparative-genomics-based tools compare the sequence of a query genome to one or more reference genomic sequences to identify elements shared by or differing between the sequences. Examples of comparative genomics software applications include IslandPick [35], which uses sequential pair-wise alignments of the query genome against each member of a group of phylogenetically related reference genomes to identify regions in the query genome not present in any of the accessory genomes, and Panseq [36, 37], which builds a nucleotide pangenome from the sequences of input genomes, then identifies presence or absence of pangenome elements in the input sequences by BLAST alignment. Limitations of currently available software for core/accessory genome analysis include restrictions on reference sequences and query file inputs to finished genomes or the inability to specifically isolate and output core and accessory sequences from query or reference genomes. The results of these and other comparative genomics based approaches will also be dependent on the selection of comparator sequences. In cases where only few poor quality and/or homogeneous reference genomes are available for comparison to the query genome, accurate and complete identification of core and accessory regions can be compromised.

Several bioinformatic tools and approaches have been developed to utilize sequence characteristics rather than comparative genomics to identify accessory elements. Many of these methods rely on the observation that horizontally-transferred elements will reflect the sequence composition of the donor genome and differ from the recipient genome. For instance, SIGI-HMM [38] identifies regions of codon usage dissimilar from comparator species and IslandPath-DIMOB [39] examines query sequence for the presence of flanking repeats, mobility genes, nearby tRNAs, and atypical dinucleotide sequence to identify genomic regions that were potentially acquired by horizontal transfer. Other tools, such as Alien_hunter [40], use variable order compositional distributions in the query genome to identify regions likely to have been horizontally transferred. These sequence-based approaches avoid some pitfalls associated with comparative genomic approaches such as choice or availability of comparator genomes. However, sequence-composition-based approaches can suffer in situations where the composition of the donor and recipient genomes is similar, as in the case of older horizontal-gene transfer events in which the sequence composition characteristics of the accessory element can come to resemble the host after sufficient generations [41]. To try to address the limitations of the comparative genomics and sequence-based approaches, some tools have been developed that combine both approaches. For instance, IslandViewer [42] combines the IslandPick, SIGI-HMM, and IslandPath-DIMOB algorithms to improve sensitivity of genomic island prediction in bacterial genomic sequences.

In this study, we propose a new comparative genomics approach to identify accessory genomic elements in draft genomes produced by NGS. We applied this approach, which addresses many of the limitations of currently available programs, to *P. aeruginosa*. We first compared twelve finished *P. aeruginosa* genomes to identify common sequences that are defined as the core genome of this bacterium. The core genome was then subtracted from the total sequences of these strains to identify their accessory genomes. Using this approach, we found that the core genome of *P. aeruginosa* was 5.84 Mbp. The accessory genome of *P. aeruginosa* accounted for 7-18% of the total genome and was enriched for genes associated with mobile genetic elements. We show the utility of this approach by applying it to two newly sequenced strains of *P. aeruginosa:* PA103 and PA99.

## Results

### Determination of the core genome of *P. aeruginosa*

**Figure 1:**
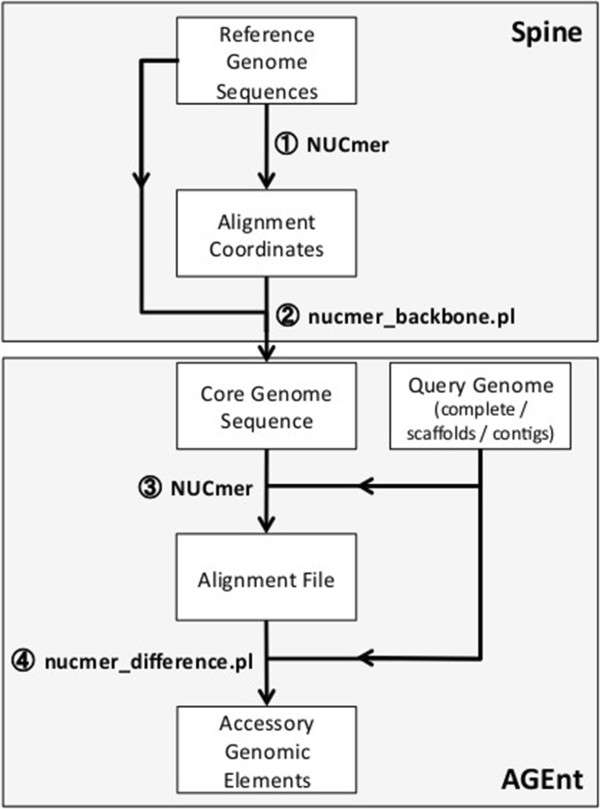
**Approach to accessory genomic element identification.** Programs used to accomplish the listed steps are indicated by circled numbers: 1, 3, NUCmer (whole-genome aligner); 2, nucmer_backbone.pl (converts coordinates of conserved regions to DNA sequence); 4, nucmer_difference.pl (subtracts regions not aligning to core). See Methods section for further details.

**Figure 2:**
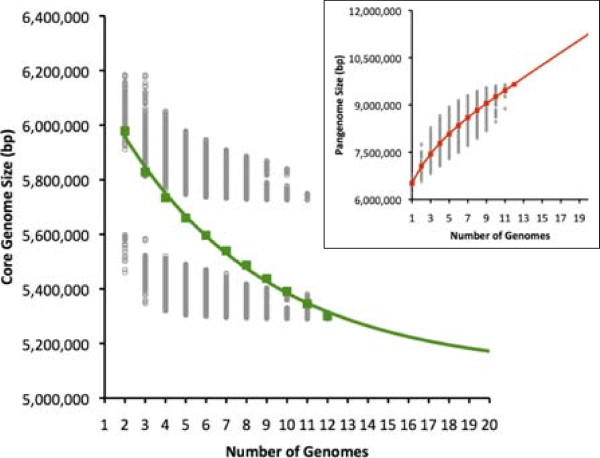
**Core genome analysis of** ***P. aeruginosa*** **.** The amount of common nucleotide sequence is plotted as a function of the number of strains sequentially added (*n*). Gray circles represent core genome size with each possible strain combination of *n* genomes. Colored squares represent the average core genome size at each *n*. The continuous curve shows the least-squares fit of an exponential decay function (R^2^ = 0.996). The inset shows the size of the nucleotide pangenome as a function of the number of strains sequentially added. The functions for both the core genome and pangenome continuous best-fit curves were derived as described in Tettelin et al. [32].

The core genome of a bacterial species consists of those sequences conserved among members of that species. To calculate the core genome of *P. aeruginosa*, we used twelve reference strains (B136-33, DK2, RP73, PAO1, PA14, PA7, LESB58, PACS2, M18, NCGM2.S1, 19BR, and 213BR) for which a single completed or mostly-completed chromosome without gaps or large numbers of ambiguous bases was available in GenBank. Only completed genomic sequences were used to calculate the core genome to minimize the potential for core sequence to be excluded as the result of undersequencing or misassembly in incomplete draft sequences. One of these strains, M18, is an environmental isolate [43], whereas the remaining eleven are human clinical isolates from diverse body sites. The genomes ranged in size between 6.26 and 6.76 Mbp (with an average size of 6.52 Mbp). To identify conserved sequences among the reference strains, we aligned the reference genomes using the NUCmer function of the MUMmer software package (Figure 1). The size of the core genome as a function of the number of reference genomes included in the analysis was calculated using an adaptation of the method described by Tettelin and colleagues [32]. As expected, the average size of the core genome decreased as more strains were included in the analysis (Figure 2). Of note, two distinct clusters of core genome size estimates were apparent, depending on which reference strains were included in the analysis (Figure 2). The cluster of smaller core genome sizes was obtained when strain PA7, a taxonomic outlier of the *P. aeruginosa* species [44], was included in the analysis. Since it is relatively dissimilar to the other PA strains, the apparent size of the core genome decreased markedly when PA7 was used to define the core genome.

**Figure 3:**
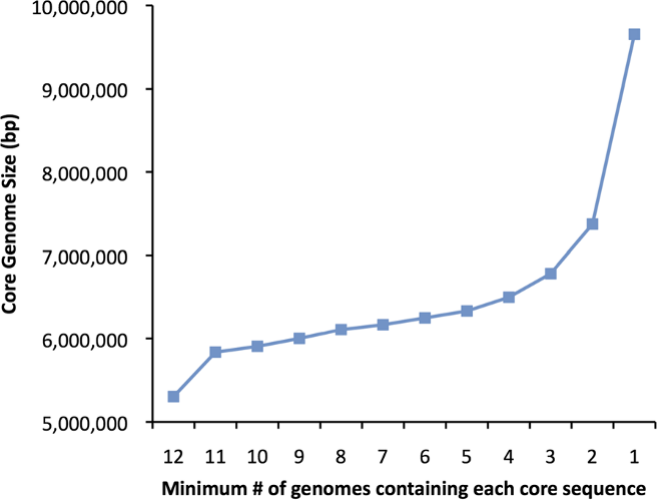
**The** ***P. aeruginosa*** **core genome size based on variable definition of “core” sequences.** The nucleotide core genome size is plotted as a function of the minimum number of the twelve reference genomes in which a particular genomic element must be present to be considered “core”. As flexibility is introduced into the definition of the core genome (i.e. an element is considered “core” if it is present in eleven of the twelve genomes, or ten of the twelve genomes, etc.), the “core” genome size increases. A “core” genome requirement of presence in only one of the twelve genomes therefore yields the pangenome of these twelve strains. Each symbol represents the average core genome size of all possible permutations of genome orders for twelve (12 permutations), eleven (132 permutations), and ten (1320 permutations) genome minimums, and the average core genome size for 10,000 randomly generated permutations at all other minimum genome numbers. Standard errors of the means at each value are too small to be visible at the scale of the figure.

The preceding analysis illustrates how inclusion of a single outlier strain can dramatically impact the size of the core genome of *P. aeruginosa*. To evaluate this phenomenon further, we calculated the core genome size based on varying definitions of the core genome: sequences shared by all strains, by at least eleven of the twelve strains, by at least ten of the twelve strains, etc. The core genome size increased substantially when sequences were allowed to be absent from one strain, but much less so as further flexibility was introduced in the definition of “core genome” (Figure 3). This substantial difference was the result of 436 kb of DNA conserved among the other eleven reference strains but absent from PA7. These sequences encode several features characteristic of the *P. aeruginosa* species such as the exotoxin A gene, *toxA*. This again suggests that inclusion of outlier strains can dramatically bias core genome size. In other words, a substantial portion of the genome that is shared by the large majority of *P. aeruginosa* isolates and therefore typical of its members may be excluded by inclusion of outlier strains. For this reason, we hereafter define the core genome as those sequences present in at least eleven of twelve (≥90%) of the *P. aeruginosa* reference genomes.

### Characteristics of the *P. aeruginosa*core genome

The DNA sequence of the *P. aeruginosa* core genome, as defined by sequences present in ≥90% of the reference genomes, was extracted from the total sequence using the script nucmer_backbone.pl, which parses the NUCmer alignment data to generate a nucleotide sequence of the core genome (Figure 1). The characteristics of the *P. aeruginosa* core genome based on these twelve reference genomes are presented in Table 1. The core genome contains 5.84 Mbp of nucleotide sequence, representing 89.7% (range: 86.4-93.3%) of the total genome. The average G + C content of the core genome is 67.0%, compared to 66.1--66.6% of the complete reference genomes. The total number of predicted genes contained within the core genome sequence is 5,316 out of 5,892 (90%) total coding sequences in PA14 and 5,570 (95%) total coding sequences in PAO1. As more completed genomes become available and are added to this analysis, it is anticipated that the size of the core genome will decrease. A curve fit to the data in Figure 2 suggests that the core genome size may plateau at approximately 5.10 Mbp, or 78% of the *P. aeruginosa* genome.

**Table 1:** ***P. aeruginosa*** **core and accessory genome characteristics**

	Core^a^	Accessory
	Avg (range)	Avg (range)
Size (kbp)	5,844	727 (430 – 1,192)
% of total genome	89.7 (86.4 – 93.3)	11.1 (6.9 – 18.0)
Average G + C %	67.0	61.2 (60.5 – 62.2)
Total # of genes^b^	5,316	608 (348 – 1,090)
Gene length (bp)	990 (72 – 13,029)	939 (51 – 17,019)
Overlapping genes^c^ (%)	28.3	33.8 (26.3 – 40.7)
Transposases^d^	7	6 (3 – 14)
Type I integron genes^e^	0	2 (0 – 10)
ICE-associated genes^f^	5	127 (4 – 192)
Predicted phage genes^g^	18	124 (19 – 271)

^a^Core genome defined as that sequence present in ≥90% of the twelve reference genomes, using PA14 as the primary reference sequence and PAO1 as the secondary reference.

^b^Number of annotated coding sequences.

^c^Percentage of coding sequences sharing at least 1 base with a neighboring coding sequence.

^d^Coding sequences with homology to proteins labeled as transposases or inactivated derivatives in COG database.

^e^Genes flanked by *sul1* and *intI1* homologs inclusive of these two genes.

^f^Genes with homologs in ICEberg protein database.

^g^Genes contained within intact, questionable, and incomplete prophage regions predicted by PHAST.

The cumulative genetic information within a bacterium’s genome is referred to as its pangenome. The size of the *P. aeruginosa* pangenome was a function of the number of reference strains included in the analysis, with each additional strain contributing further genetic information to the pangenome (Figure 2, inset). Thus, as observed by others [45], *P. aeruginosa* has a theoretically open genome (i.e. the pangenome will continue to increase in size with each additional strain sequenced). Extrapolation of the best-fit line of the average pangenome size as a function of genome number suggests that each additional sequenced strain will yield 197 kb of novel genetic information. Since our best-fit line is based on only 12 strains, it is likely that this estimate will need to be revised as additional genome sequences become available.

### Characteristics of the *P. aeruginosa*accessory genome

**Figure 4:**
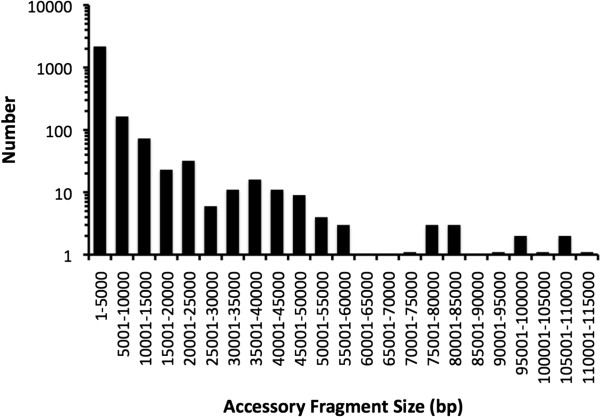
**Sizes of accessory genomic regions.** The size distribution of all accessory genomic elements identified among the twelve reference genomes are plotted. For each of the ranges 7001-7500 bp, 9001-9500 bp, 100001-10500 bp, and 110001-11500 bp, only 1 element was found, as indicated in the figure by a bar of artificially non-zero height on the log scale.

Following extraction of the core genome sequences, the remaining sequences of the twelve reference strains were designated the accessory genome. Since *P. aeruginosa* has a theoretically open genome [45], these sequences in actuality represent only a small sampling of the entire *P. aeruginosa* accessory genome, but nonetheless provide insight into the features of these sequences. The average size of the accessory genome of a *P. aeruginosa* strain was 727 kbp, representing 11.1% (range: 6.9-18%) of the total genome (Table 1). Accessory genome G + C content was lower than that of the core genome, averaging 61.2% for the twelve reference strains. Among the reference genomes examined, an average of 608 genes (range: 348-1,090) were contained in the accessory genome of each strain. Although the majority of accessory genomic segments are smaller than 5 kb, contiguous accessory elements >100 kb in size were identified (Figure 4).

### Comparison of the core and accessory genomes

The accessory genome was enriched for a number of mobile genetic elements [16] (Table 1). Sequences with homology to ICE and phage genes were concentrated in the accessory portions of the reference *P. aeruginosa* genomes. In addition, several sequences associated with type 1 integrons were found in the accessory genomes of the strains, but none were present in the core genome. Putative transposases were found in equal numbers within both the accessory and core genomes; those within the core genome may represent ancient and non-functional genes. These results confirmed that sequences associated with mobile elements were concentrated in the accessory genome of *P. aeruginosa* and further validated our method of separating core and accessory genomes.

**Figure 5:**
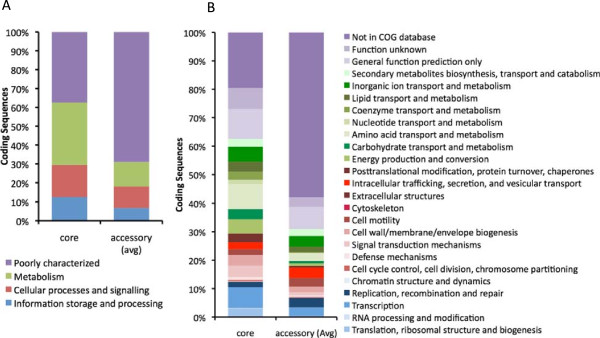
**Functional annotations of core and accessory genes. (A)** COG categories and **(B)** COG subcategories of predicted genes within the core and accessory genomes of *P. aeruginosa*. Each category or subcategory is graphed as a percentage of the total number of genes in the core or accessory genomes. Accessory genome percentages are averages of the twelve reference strains.

Coding sequences belonging to the core and accessory genomes were assigned to putative super-functional (Figure 5A) and functional (Figure 5B) categories using the Clusters of Orthologous Groups of proteins (COG) database [46, 47]. Approximately one-third of the predicted genes in the core genome were dedicated to metabolic functions. One-third was roughly evenly split between cellular process/signaling functions and information storage/processing functions. Functions of those predicted genes in the remaining third of the core genome were either poorly categorized or uncategorized. In the accessory genomes of the reference *P. aeruginosa* strains, on the other hand, more than two-thirds of the accessory genes fell into the categories of poorly characterized or uncharacterized functions, and almost half had no identifiable ortholog in the COG database (Figure 5B).

**Figure 6:**
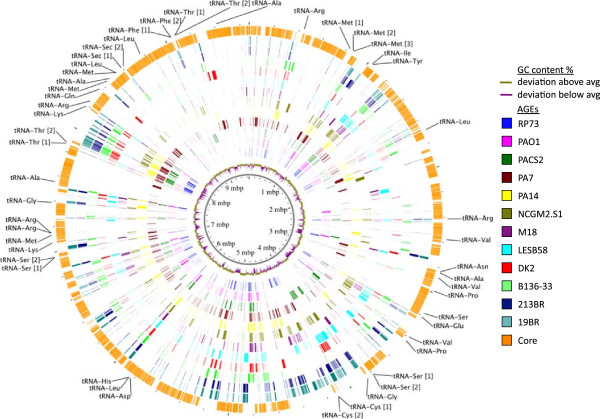
**Pangenome and core genome of twelve strains of** ***P. aeruginosa*** **.** The inner plot shows the deviation of GC content of each region above or below the mean GC content of the pangenome. Colored rings show accessory genomic elements of each reference strain. The outer orange ring shows the distribution of core genomic elements along the pangenome. PA14 tRNA gene locations are indicated with tRNA gene names followed by numbers in square brackets in cases of gene interruption by accessory sequence. (Figure format was adapted from Mathee et al. [12]).

We next examined the architecture of the pangenome of the twelve reference *P. aeruginosa* genomes and the relative locations of the core and accessory genomic elements. As has been shown previously [12], accessory elements were more concentrated in some regions of the chromosome than others (Figure 6). However, the overall distribution of accessory elements was actually quite broad. The core genome derived from the twelve *P. aeruginosa* reference strains consisted of 884 segments 10 bp or larger and was interrupted on average every 6.6 kb by an accessory element (data not shown). The largest uninterrupted stretch of core genome consisted of 82 kb of sequence (data not shown).

### A method for rapidly identifying accessory genomic elements in newly-sequenced *P. aeruginosa*strains

The theoretically open genome of *P. aeruginosa* implies that novel accessory genomic elements are likely to be present in each newly sequenced strain. As additional strains of *P. aeruginosa* are sequenced, a method for rapidly identifying their AGEs will be necessary. Here we describe AGEnt, an algorithm developed for this purpose (Figure 1). AGEnt computationally subtracts previously derived core genome sequences from a query draft or complete genome sequence to yield the accessory genome. The program has two inputs: 1) the sequence of a query genome as contigs, scaffolds, or completed chromosome(s) and 2) a core genome sequence. It outputs sequences that belong to the accessory genome of the query strain [48].

To test the fidelity of this approach, we applied it to each of the twelve reference strains and compared its results to the accessory genome sequences previously generated by multiple alignments (Table 1). Only predicted elements with a minimum length of 10 bases were included. Comparison of accessory element sequences in each genome identified by AGEnt with those identified in the preceding analysis (“non-core”) showed that in all cases agreement was good (Additional file 1: Table S1). Nearly all (99.06%) of the non-core sequence was also identified by AGEnt. The amount of genomic sequence identified by AGEnt that was not found in the non-core sequence set was also very low, ranging between 0.01% and 0.30% (average 0.20%) of the total amount of AGE sequence identified. The taxonomic outlier PA7 showed the most discrepancy between the AGEnt output and previously identified non-core sequence. Thus the AGEnt algorithm accurately identifies accessory genomic sequences.

**Figure 7:**
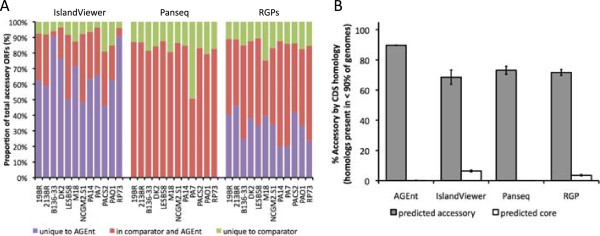
**Performance of AGEnt compared to other predictors of** ***P. aeruginosa*** **accessory genome.** AGEnt was compared to IslandViewer, Panseq, and RGPs for identification of accessory genome sequences. **(A)** Colored bars represent the proportion of accessory genome coding sequences (CDS) identified by both AGEnt and the comparator method (red), identified by AGEnt but not the comparator method (purple), or identified by the respective comparator method but not AGEnt (green). **(B)** Evaluation of the accuracy of accessory genome identification by AGEnt and the comparator methods using gene homology searches as a gold-standard. Genes were considered homologous if they shared at least 50% sequence identity across at least 50% of the gene length. CDSs in each of the genomes were classified as accessory if they were found to have homologs in <90% of the twelve reference genomes by sequential sequence alignments (see Methods for details). Bars represent average percentage of total CDS called by each method as accessory (grey bars) or core (white bars) subsequently identified as accessory by gene homology.

To further evaluate its performance, AGEnt was compared to other algorithms for identifying accessory genome. These included the following: 1) IslandViewer [42], a web-based application that combines three algorithms for identifying potential genomic islands in bacterial sequences using whole-genome alignments with reference strains, sequence composition, codon usage characteristics, and homology with known mobility genes, and 2) the Pan-Genome Analysis module of Panseq [36], which uses BLASTn and NUCmer to evaluate for presence or absence of genomic segments among a set of user-provided bacterial genome sequences. The AGEnt algorithm identified substantially more coding sequences in each reference *P. aeruginosa* strain as accessory than did IslandViewer (Figure 7A). The amount of sequence identified as accessory by both of these methods was relatively small. In contrast, most of the sequences identified as accessory elements by Panseq were also identified as accessory by AGEnt (Figure 7A). Panseq did, however, also identify a large amount of sequence as accessory that was not identified as such by AGEnt.

Another method to identify accessory genome sequences relies upon genomic context. Mathee and colleagues compared five sequenced *P. aeruginosa* strains to identify clusters of genes not shared among all five genomes [12]. The authors identified 52 such regions they designated as Regions of Genomic Plasticity (RGPs), which were defined as polymorphic strain-specific segments including at least four open reading frames (ORFs) flanked by conserved “anchor” ORFs and missing in at least one of the genomes analyzed [12]. As more genome sequences became available, further *P. aeruginosa* RGPs were described [44, 49–51]. Comparison of genes contained within RGP borders against coding sequences in the accessory genome identified by AGEnt revealed that an average of 39% of genes in accessory sequence called by AGEnt were not contained within described RGP borders (Figure 7A).

To further evaluate the accuracy of AGEnt relative to the other methods of identifying accessory genome, we used homology searches to designate genes (rather than sequences) as core or accessory. All annotated coding sequences in each strain were screened to determine whether homologs of each gene were present in the other reference strains. A minimum of 50% sequence identity across at least 50% of the gene length was defined as a gene homolog. For the purposes of analysis, genes found to have homologs in ≥90% (11 or 12) of the 12 reference strains were considered to be core genes, and genes found to have homologs in <90% (≤10) of the reference strains were considered accessory genes. All genes designated as accessory by each of the prediction methods (AGEnt, IslandViewer, Panseq, or RGPs) were categorized as core or accessory by gene homology in this fashion (Figure 7B). An average of 89.7% of genes predicted to be within the accessory genomes of the reference strains by AGEnt were also found to belong within the accessory genomes by gene homology, versus 68.5% of accessory genes predicted by IslandViewer, 73.1% of genes predicted by PanSeq, and 71.6% of genes predicted by RGP analysis (p < 0.001 in comparison to AGEnt in all three cases). Genes that were not found within the accessory genome in each method were also evaluated. Less than 0.1% of genes predicted to be core by AGEnt were not predicted to be core by gene homology, versus 6.4% (p < 0.001), < 0.1% (p = 0.27), and 3.6% (p < 0.001) of genes identified as core by IslandViewer, PanSeq, and RGP analysis, respectively (Figure 7B). When a more conservative definition of gene homology was used (at least 80% sequence identity across at least 80% of the gene length), predicted accessory genes determined to also be accessory by gene homology increased to 99.2% for AGEnt, but concordance among the other prediction methods remained significantly lower at 72.0%, 82.3%, and 78.2% for IslandViewer, PanSeq, and RGP analysis, respectively (p < 0.001 for all three comparisons to AGEnt). These results suggest that AGEnt successfully identifies genomic regions containing genes that are accessory to the *P. aeruginosa* genome and that few potential accessory genes are being missed by this method.

### Identification of accessory genomic elements in newly-sequenced *P. aeruginosa*genomes

Since most newly sequenced bacterial genomes are released as draft (as opposed to finished) sequences, we evaluated the utility of AGEnt in the identification of the accessory genome from draft sequences. In particular, we applied it to draft sequences of two commonly used but previously unsequenced strains of *P. aeruginosa*, PA99 and PA103. PA99 is a clinical isolate obtained from the urine of an adult patient [52]. This strain is characterized by secretion of the three type III secreted effectors ExoS, ExoT, and ExoU [53]. Most strains of *P. aeruginosa* secrete either ExoS or ExoU but not both [52]. PA99 has been used extensively to study the *P. aeruginosa* type III secretion system [54–56]. PA103 was first isolated from the sputum of a patient [57] and has been frequently used in studies of *P. aeruginosa* virulence [25, 58–60]. Sequencing of both strains was performed on the Illumina HiSeq 2000 platform generating 18.30 and 12.65 million paired-end 100-bp reads for PA99 and PA103, respectively. *De novo* assembly of the draft genome sequences was performed using Ray assembly software [61] and annotation was performed with the RAST automated annotation server [62]. Assembly and annotation characteristics of both genomes are found in Table 2.

**Table 2:** **Genome characteristics of** ***P. aeruginosa*** **strains PA99 and PA103**

	PA99	PA103
# of contigs	142	270
Total contig size (bp)	6,343,993	6,716,795
Contig N50 (bp)	92,875	53,587
Average contig G + C (%)	66.1	66.1
Predicted CDSs (#)	5,739	6,103
Accessory genome size (bp)	688,597	918,208
% of total contigs size	10.8	13.7
Average accessory G + C (%)	59.9%	60.8%
Accessory ORFs (#)	493	732
Transposases^a^	2	1
Type I integron genes^b^	0	0
ICE-associated genes^c^	101	216
Predicted phage genes^d^	25	69

^a^Coding sequences with homology to proteins labeled as transposases or inactivated derivatives in COG database.

^b^Genes flanked by *sul1* and *intI1* homologs inclusive of these two genes.

^c^Genes with homologs in ICEberg protein database.

^d^Genes contained within intact, questionable, and incomplete prophage regions predicted by PHAST.

**Figure 8:**
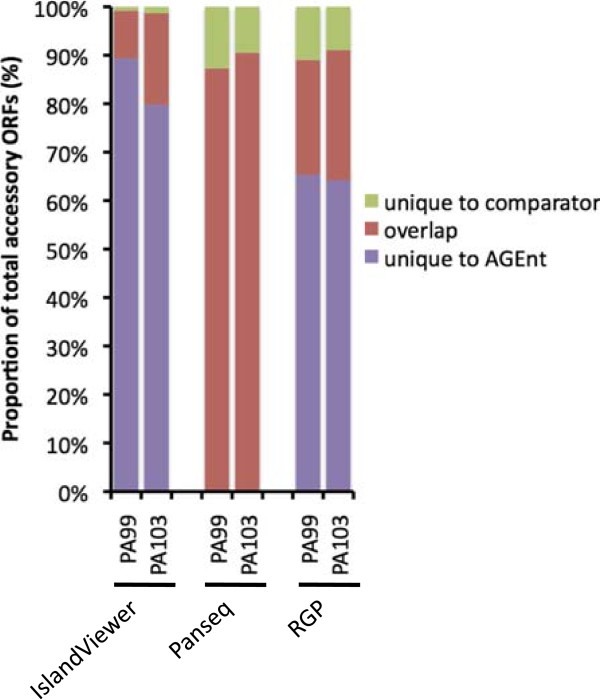
**Performance of AGEnt in identifying accessory sequences in the draft genomes of** ***P. aeruginosa*** **strains PA99 and PA103.** AGEnt was compared to IslandViewer, Panseq, and RGPs for identification of accessory genome sequences. Colored bar sections represent the proportion of accessory genome CDS identified by both AGEnt and the comparator method (red), identified by AGEnt but not the comparator method (purple), or identified by the respective comparator method but not AGEnt (green).

PA99 and PA103 accessory genomes were identified by AGEnt using the core genome derived from the 12 reference strains of *P. aeruginosa*. The accessory genome of PA99 was 688 kb in size and contained 493 ORFs (Table 2). The accessory genome of PA103 was 918 kb in size and contained 732 ORFs (Table 2). Similar to the reference strains, the accessory genomes of PA99 and PA103 were enriched for transposases, ICE genes, and phage-associated genes, whereas no type I integron genes were found in either PA99 or PA103 (Table 2). The performance of AGEnt on these draft genomes was compared to other methods for determining the accessory genome. As with the reference genomes, a large number of ORFs that AGEnt designated as accessory were not also identified as accessory by IslandViewer or RGP analysis (Figure 8). Similar to the pattern of Panseq identification of accessory genome in the reference strains, there was a greater overlap of ORFs called by both AGEnt and Panseq in PA99 and PA103, but several more ORFs identified by Panseq were not also called accessory by AGEnt (Figure 8). To evaluate the accuracy of AGEnt, the ORFs of the accessory genomes of PA99 and PA103 were compared to the coding sequences of the twelve reference genomes by BLAST analysis. Using the criteria of at least 80% sequence identity across at least 80% of the gene length, more than 98% of the ORFs in PA99 and more than 99% of the ORFs in PA103 identified as accessory by AGEnt were found to have homologs in <90% of the 12 reference strains. By contrast, fewer than 1% of the coding sequences in either strain that were not contained in the identified accessory regions had homologs in <90% of the reference strains. These results indicate that AGEnt can effectively distinguish accessory genomic elements from core elements in draft genomes of *P. aeruginosa* produced by NGS.

## Discussion

Comparative genomic studies have demonstrated that the accessory genomes of bacteria play important roles in niche adaptation and virulence. It is anticipated that continued study will result in descriptions of core genomes that are progressively better characterized and defined, but novel accessory genomic sequences will continue to be forthcoming, especially in bacteria such as *P. aeruginosa* with theoretically open genomes. For this reason, methods for rapidly and accurately identifying accessory genome sequences are needed. To accomplish this, we developed a new software algorithm for identification of both core and accessory genomes from bacterial genome sequences. First, Spine determines the “core” genome from a group of genomic sequences. This information is then used in conjunction with AGEnt to identify accessory genome in draft genomic sequences. Here we applied this approach to the opportunistic pathogen *P. aeruginosa*. Comparison to results generated by previously published programs indicated that AGEnt performed well and is capable of accurately identifying accessory genome sequences in draft genomes produced using NGS technology.

In our analysis, the *P. aeruginosa* core genome was found to be 5.84 Mbp in size, containing 5,316 genes. These results are based on a ‘soft’ definition of core genome as consisting of those regions present in ≥ 90% of the reference strains. Previous studies had found the *P. aeruginosa* core genome to contain 4,934 – 5,021 genes [12, 51] representing roughly 86% to 88% of the total *P. aeruginosa* genome. These earlier analyses, however, were based on fewer genome sequences (4 – 5) and used a ‘strict’ definition of core genome, including only genes that were present in 100% of strains. In our analysis, the accessory genome of *P. aeruginosa* was found to comprise 11% of the total genome size, which compares well to previous estimates of approximately 10% [12, 63, 64]. Therefore, the accessory genome of *P. aeruginosa*, as a proportion of the total genome size, is smaller than that of some other pathogenic bacteria, such as *E. coli* (20% [65]), *Acinetobacter baumannii* (12 – 19% [66]), *Listeria monocytogenes* (12 – 23% [67]), and *Staphylococcus aureus* (25% [68]), but larger than other bacteria with closed pangenomes, such as *Bacillus anthracis* [69] and *Francisella tularensis* [70].

Our approach to identifying a strain’s accessory genome offers several advantages over other methods. By defining the *P. aeruginosa* core genome based on conserved sequence rather than conserved genes, potential bias introduced by gene-calling was eliminated and non-coding regions were also included in the defined core genome [71]. This is advantageous, as non-coding elements such as small RNAs are increasingly being recognized as important in the physiology of *P. aeruginosa* [72]. By circumventing the requirement for annotation, this approach allows for identification of accessory genome sequences from newly sequenced strains in minutes through a web application or on a desktop computer. As NGS whole-genome sequencing technology becomes more widespread, easily accessible, and affordable, researchers are able to quickly generate draft sequences of dozens of bacterial strains in a single sequencing run. As a result, the number of draft genomes being generated is far outpacing “finished” genomes with complete annotations [73]. Because of the shorter sequencing reads generated by NGS technologies and the corresponding inability to unambiguously resolve longer nucleotide repeat structures within the genome, these draft genomes contain many gaps [74]. The ability to easily and accurately identify the accessory genomic sequences from these draft sequences without slow and computationally demanding cross-comparisons of coding sequences to group homologous proteins will become increasingly important in comparative genomic studies. Such analyses would be of particular utility in studies of the contribution of accessory elements to phenotypes such as virulence, antibiotic resistance, or metabolic diversity. Given that some complex phenotypic characteristics such as virulence have been shown to be combinatorial in *P. aeruginosa* [75], the complete capture of a strain’s accessory genomic content will be of considerable importance to future studies correlating *P. aeruginosa* phenotypes with specific accessory genomic elements.

Compared to other currently available applications, Spine/AGEnt shows improved accuracy and flexibility. In the case of IslandViewer, some reasons for the discrepancies observed may include IslandViewer’s potential to miss smaller accessory regions since islands smaller than 3 kb are not included in the results, and only a limited number of reference genomes for comparison are available in the site’s online database. In addition, the IslandPick application in IslandViewer excludes as GIs regions that are found in any of the comparator reference genomes. Thus this method is best suited for identifying novel accessory sequences that are unique to the query strain and have not been previously observed in the reference strains. Finally, as some of the IslandViewer methods rely on identifying typical architectural characteristics of GIs to call accessory regions, the input of randomly scaffolded contigs from the draft assemblies of PA99 and PA103 could conceivably have resulted in reduced sensitivity for identifying accessory elements.

There are more similarities between the approaches taken by Panseq and Spine/AGEnt. Like AGEnt, Panseq does not require the input sequences to be either complete or annotated. The software, however, is not as directed at defining core or accessory sequences or genomic coordinates from input genomes and therefore some post-hoc manipulation of the standard Panseq output was necessary for comparison to AGEnt. The standard output by the web version of Panseq provides the alignment coordinates of each of the reference genomes against the pangenome sequence rather than against the input sequences. To allow direct comparison of the performance of Panseq to AGEnt, the additional step of converting these pangenome alignment coordinates to accessory coordinates (i.e. the coordinates of regions in each strain aligning to <90% of the reference strains) was necessary (see Methods). The Panseq analysis results were also dependent upon the values used for several adjustable parameters. For our comparison, we used the web version of Panseq’s default settings: 500 bp minimum novel region size, 500 bp fragmentation size, and 90% sequence identity cutoff. It is conceivable that Panseq could perform comparatively better against Spine/AGEnt with different parameter settings. However, using settings of 50 bp minimum novel region size, 50 bp fragmentation size, and 85% sequence identity cutoff, we found even more core genome sequence was misidentified as “accessory” by Panseq (data not shown).

As described above, regions of genomic plasticity (RGPs) represent locations in the *P. aeruginosa* genome where accessory elements are likely to be found, but our results suggest that examining only these sites in a given strain may miss almost half of accessory genomic sequences. Relying on defined insertion “hotspots” in the *P. aeruginosa* genomes suffers from the limitation that novel insertion sites may only become apparent as more sequences are examined [76]. Thus it is anticipated that the accuracy of this approach will improve as more genome sequences become available. It is also apparent from our results that a non-trivial percentage of coding sequences within RGPs do not belong to the accessory genome (Figure 7B). This may be explained by RGPs having been defined from a smaller group of reference genomes and absence or deviation in content from even one of the genomes was sufficient to warrant definition as an RGP. Another explanation for this finding may be that certain genes are found between different RGP anchors in different strains, yet are present in most or all *P. aeruginosa* strains. The RGP approach would define such genes as accessory. In contrast, AGEnt, which does not take location of genes in the genome into account when defining accessory versus core would label these genes as core. This minimizes false calls in cases of genome rearrangements or mosaic accessory elements.

Our analysis and comparison of Spine/AGEnt to other bioinformatic tools was not exhaustive. Other than the *P. aeruginosa*-specific RGP method, we tried to choose functional web-based applications representative of both comparative genomic and sequence-based approaches to accessory genome identification. We did not evaluate some other applications available for identifying accessory sequences in bacterial genomes that made use of similar approaches. Examples include, but are not limited to, MobilomeFINDER [77], Mosaic [78, 79], mGenomeSubtractor [80], PIPS [81], and Alien_hunter [40].

The Spine/AGEnt approach to identifying accessory genomic elements in *P. aeruginosa* has certain limitations. Whole-genome alignment of reference genomes and core genome identification with Spine can be time-consuming with standard computer resources. We have tried to minimize this bottleneck by designing Spine to run analyses in parallel processes when computing hardware allows. However, once the step of core genome definition by Spine has been performed, subsequent backbone subtraction with AGEnt is a much more rapid process, requiring seconds or minutes to complete. A second limitation is that only 12 genomes were used to define the *P. aeruginosa* core genome. As more *P. aeruginosa* genomes are sequenced and finished, updating of the core genome definition by adding these new genomes to the whole-genome alignment will undoubtedly reassign some sequences currently defined as core to the accessory genome and vice versa. For this reason, we have designed both Spine and AGEnt with the flexibility to accept any user-defined set of genome sequences. This will allow for easy updating of the core sequence definition as more completed genomes become available.

We defined the core genome as sequences present in ≥90% (at least 11 of 12) of reference genomes. We chose this less restrictive definition rather than requiring that core sequences be present in all members of the species [69] for several reasons. Although such a set of “core” genes may contain genes that are absent in a few members of the species, it will nonetheless capture genes that define the vast majority of strains in the species and might reasonably be considered typical of the species. This approach prevents a few outlier strains from dramatically biasing the set of core genes characteristic of a bacterial species. For example, the major exotoxin gene *toxA* and the quorum sensing regulator *mvfR* are commonly found in *P. aeruginosa* strains but are missing from strain PA7 [44]. A strict definition of *P. aeruginosa* core genome would exclude these genes from the core genome of *P. aeruginosa* despite their near ubiquitous presence and prominent role in defining the characteristics of the species. The 90% threshold also makes the core genome less sensitive to future reclassification of a minor subset of strains to different species. It has been proposed that as more genome sequences become available, bacterial species classification will become more defined by genome characteristics and less by DNA-DNA hybridization patterns [82]. If this is indeed the case, strains such as PA7 may one day be excluded from the *P. aeruginosa* species. These concerns are not unique to *P. aeruginosa* but have also been observed in other species [83]. For this reason, we feel a less restrictive definition of the *P. aeruginosa* core genome, as used in this study, has better utility in defining the core genome of a bacterial species. As the number of strains used to define core genome increases with future sequencing projects, a more rigorous definition of core (i.e. present in ≥ 95% of strains) may provide a better balance between over- and underestimating the true species core genome.

A limitation of comparative genomics approaches to core/accessory definition, including Spine/AGEnt, is that the selection of reference sequence(s) for comparison can bias the output. In this study, for example, eleven of the twelve finished *P. aeruginosa* genomes available to serve as reference genomes were clinical isolates obtained from infections, Therefore it is conceivable that some genomic regions determined to be “core” by this analysis may be common to clinical strains but not represented in environmental strains. The identification of a more generally species-specific core genomes will likely be facilitated by inclusion of a greater number of genomic sequences from a diversity of sources as they become available. Additionally, combination of the results of Spine/AGEnt with a sequence-based approach to identifying horizontally transferred genetic elements, such as Alien_hunter [40], SIGI-HMM [38], or IslandPath-DIMOB [39], could also potentially increase accessory element detection sensitivity through examination of sequence composition characteristics and/or mobility gene locations that would be independent of any comparator reference genome(s).

Definition of a representative species core genome sequence could assist in assigning newly sequenced strains to a species. Bacterial species definitions have been subject to debate [82] and several definitions have been introduced. For example, Stackebrandt & Goebel proposed that two strains belong to a species if they share 97% or more sequence similarity in their 16S rRNA genes [84]. Others have proposed that average nucleotide identity (ANI) of less than 95% in the alignment of two whole-genome sequences can be used to differentiate species [85, 86]. Previous reports have suggested a role for using core genome contents to define taxonomic boundaries [87, 88]. Core genome gene alignments were used to show that *Azobacter vinelandii* clusters closely with other *Pseudomonas* species, suggesting it may belong to the *Pseudomonas* genus [89]. Core genome sequence analyses were used in other recent genomic studies of *P. aeruginosa* to define strain relatedness [90, 91]. A species majority core genome, such as that identified by Spine, could serve as a basis for assigning newly sequenced strains to species. Similar to an ANI calculation between two strains, a strain could be assigned to a species based on the total amount of the core genome it contained and a threshold for the amount of core genome in common could be established, below which a strain would be considered to belong to a different species. Further analyses and genome comparisons would be required to evaluate the feasibility of such an approach to taxonomic designation.

Although this report focuses on characteristics of the *P. aeruginosa* core and accessory genomes, the methods described can be generalized to other prokaryotes. As long as multiple complete, finished genomes of the organism of interest are available, it will be possible to define a core genome and to apply it to derive accessory genomic sequence from query genomes using these tools. Indeed, Spine also allows inclusion of incomplete or draft genomes when generating the core genome sequence although at the possible expense of some specificity in identifying the accessory genome. Further studies using Spine and AGEnt to identify the accessory genomes of other bacterial species are ongoing.

## Conclusions

We have developed new software tools, Spine and AGEnt, to identify core and accessory genome from nucleotide sequences of finished or draft genomic sequences. We have used this software to determine a *P. aeruginosa* core genome from the sequences of twelve reference genomic sequences and used this to define the accessory genome of two newly sequenced strains, PA99 and PA103. Spine and AGEnt compared favorably to other methods for differentiating core and accessory genome in *P. aeruginosa*. This study increases our understanding of the composition and characteristics of the *P. aeruginosa* genome as well as provides new tools for studying the variable component of bacterial genomes.

## Methods

### Reference strains, mobile element prediction, and whole-genome sequencing

The annotated complete genome sequences of *P. aeruginosa* strains B136-33 (CP004061.1), DK2 (CP003149.1), LESB58 (FM209186.1), M18 (CP002496.1), NCGM2.S1 (AP012280.1), PA7 (CP000744.1), UCBPP-PA14 (CP000438.1), PACS2 (NZ_AAQW01000001.1), PAO1 (AE004091.2), and RP73 (CP006245.1) were obtained from NCBI GenBank. The nucleotide sequences of 19BR (AFXJ01000001.1) and 213BR (AFXK01000001.1), which were not yet annotated in GenBank, were downloaded and automated annotation performed using the Rapid Annotations using Subsystems Technology (RAST) web service [62]. Functional annotation of genes and transposase identification was accomplished by BLASTp alignment of annotated ORFs against the COG database [46, 47] using BLAST + v2.2.24 [92, 93]. Prophage sequence was predicted in the reference strains using the web-based service PHAST [94], which detects prophage sequences in bacterial genomes using database comparisons and feature identifications. ICE genes were identified by BLAST homology to proteins in the ICEberg database [95] using homology cutoffs of Evalue ≤1e-6 and percent identity ≥ 85%. To identify integron sequences, the type 1 integron flanking sequences of *sul1* and *intI1* from NCGM2.S1 were obtained from the *Pseudomonas* Genome Database [96]. Nucleotide BLAST alignment of these sequences against the reference genomic sequences was used to identify potential type 1 integron structures.

Genomic sequencing of the *P. aeruginosa* strains PA103 and PA99 was performed on the Illumina Hi-Seq 2000 platform by the Genomics Resource Center at the University of Maryland School of Medicine Institute for Genome Sciences with 100 bp paired-end reads yielding at least 100-fold average read coverage across each strain. The reads were *de novo* assembled using Ray v2.0.0-rc5 [61] and resulting contigs annotated using RAST. Open reading frames smaller than 50 amino acids were discarded and annotations manually edited as needed. The Whole Genome Shotgun projects have been deposited at DDBJ/EMBL/GenBank under the accessions JARJ00000000 (PA99) and JARI00000000 (PA103). The versions described in this paper are versions JARJ01000000 (PA99) and JARI01000000 (PA103).

### Core genome identification

For the purposes of this study, the core genome was defined as those sequences present in nearly all genomes from bacteria of a given species. Spine, a program wrapper written in Perl was developed to identify core genome from genomic DNA sequences (Figure 1). The software is available as a web-based application or for download as a command-line script [48]. Spine identifies core genome sequences by first performing genome alignments of user-supplied reference strain sequences using the NUCmer function of the MUMmer software package v3.23 [97, 98]. An all-vs.-all alignment of the reference strains is performed using the “--maxmatch” option to preserve all unique and non-unique matches. Otherwise default NUCmer parameters are used. The resulting alignment file is converted to alignment coordinates and sorted by reference ID using MUMmer’s “show-coords” function. Spine then outputs DNA sequence and genomic coordinates of regions present in a user-defined subset of the reference genomes using the NUCmer alignment coordinate file and the sequences of the reference genomes. For this study of twelve *P. aeruginosa* reference genomes, only alignments with at least 85% sequence identity were considered homologous. Note that this analysis allows for and includes as core those conserved sequences that are duplicated or repeated in certain strains. Spine also outputs accessory genome sequences and their genomic coordinates (see below). An individual annotated *P. aeruginosa* gene was categorized as core if ≥ 50% of the nucleotide sequence of that gene was contained within the core coordinate set or as accessory if > 50% of the gene sequence was contained within the accessory coordinate set. For the core genome description, sequence and gene definitions from the annotated PA14 genome were used primarily, i.e. genomic sequence regions in PA14 found to have homologous regions in at least 10 of the other reference strains and the annotated genes in those PA14 regions were used to define most of the core sequence. PAO1 genomic sequence was used for core regions found in 10 of the reference genomes, but not PA14.

The alignments generated by Spine were also used to estimate nucleotide core genome and pangenome size based on an adaptation of a method described by Tettelin et al. [32]. Briefly, all possible combinations of sequential inclusion of up to twelve reference strains were evaluated to determine the impact on the amount of conserved sequence present in the included genomes (“core genome”) and the amount of unique sequence among the included genome sequences (“pangenome”). Locations of core and accessory genomic regions in the *P. aeruginosa* pangenome were plotted using the program CGView [99].

### Accessory genome prediction

For the purposes of this study, the accessory genome was defined as those sequences found in some *P. aeruginosa* strains but not others (i.e. all sequences not part of the core genome). Accessory genome prediction in bacterial genomic sequences was performed using the algorithm AGEnt, which relies on a combination of the NUCmer function of the MUMmer software package v3.23 [97, 98] and our Perl script nucmer_difference.pl (Figure 1). Briefly, NUCmer is used to create an alignment of the query genome against the core genome sequences generated as described above. The resulting “delta” alignment file is used as input for nucmer_difference.pl, which identifies the genomic coordinates of regions not aligning to the core genome. The nucmer_difference.pl script then produces a table of coordinates of non-core regions in the query genome and the nucleotide sequences of these regions. If provided with a list of gene coordinates, nucmer_difference.pl will also output the genes within these regions that are part of the accessory genome. AGEnt is implemented in Perl and is available as both a web-based application and for download [48]. Output parameters are customizable, but for these analyses, an individual gene was considered to belong to the accessory genome if ≥ 50% of its sequence was contained within the coordinates of an accessory region.

Genomic islands in the genomic sequences were predicted using the IslandViewer web service [42]. The “pan-genomic sequence analysis” function of PanSeq [36, 37] was run on the twelve reference genomes using default parameters except that “coreGenomeThreshold” was set at 11. To allow the results of the Panseq analysis to be directly compared to AGEnt results, the “pan_genome.txt” output file was used to determine coordinates of all reference sequences present in less than eleven of the twelve input strains. *P. aeruginosa* regions of genomic plasticity (RGPs) were identified in the reference strains based on nucleotide BLAST homologies to flanking coding sequences of those RGPs summarized by Klockengether et al. [51]. Only RGPs containing at least one and no more than two hundred coding sequences in a particular strain were included in the analysis. For IslandViewer, accessory regions were predicted in unfinished, draft genomes by first concatenating scaffolds, separating each scaffold with a string of 20 ambiguous bases. Accessory regions of draft genomes were predicted in Panseq by first producing a core genome sequence consisting of regions present in at least eleven of twelve reference genomes from the “pan_genome.txt” output file, as above. This core genome sequence was then uploaded as “reference” and the draft genome sequence as “query” in the “Novel Region Finder” module on the Panseq site. For RGP analysis of draft genomes, only RGPs with anchors on the same scaffold were included as accessory genome.

To compare the performance of the AGEnt algorithm to conventional definitions of core and accessory genomes that rely on the presence or absence of discrete genes (as opposed to sequences), classification of individual coding sequences as being present or absent in each genome was determined by gene sequence homology as previously described by Kittichotirat et al. [34]. Briefly, coding sequences in each query genome were compared to the twelve reference genomes by four separate alignments: i) comparison of the DNA sequence of the query gene to all gene sequences in the reference genomes; ii) comparison of the DNA sequence of the query gene to the genomic DNA sequences of the reference genomes; iii) comparison of the protein sequence of the query gene to all protein sequences in the reference genomes; and iv) comparison of the query protein sequence of the query gene to all translated proteins from the genomic DNA sequences of the reference strains. BLASTn was used to produce alignments in steps i and ii. tBLASTn was used to produce alignments in step iv. The ublast function of usearch v6.0.307 [100] was used in step iii for improved speed over BLASTp. To allow sufficient specificity for identifying genes belonging to the accessory genome, alignment hits with at least 50% sequence identity and at least 50% sequence coverage between query and hit sequences in one or more of the analysis steps were considered homologous. If a homolog to a particular gene was found in at least eleven of the twelve reference genomes, it was considered a “core” gene, whereas genes with potential homologs in less than eleven of the twelve reference genomes were classified as “accessory” genes. Statistical analysis of the means was performed using the Student’s t-test.

## Availability of supporting data

The software developed for this study can be found for online use (Spine, AGEnt) or download at http://vfsmspineagent.fsm.northwestern.edu.

## Electronic supplementary material

Additional file 1: Table S1: Prediction of accessory elements in reference *P. aeruginosa* genomes using AGEnt and comparison to regions excluded from core genome generation in each reference strain. (DOC 42 KB)
