# Supplementary material for: Characterization of the core and accessory genomes of Pseudomonas aeruginosa using bioinformatic tools Spine and AGEnt
Source: BMC Genomics. 2014 Aug 29;15(1):737. doi: 10.1186/1471-2164-15-737 (PMC4155085; doi:10.1186/1471-2164-15-737)
Supplement: Supplementary file 1 — Additional file 1: Table S1: Prediction of accessory elements in reference P. aeruginosa genomes using AGEnt and comparison to regions excluded from core genome generation in each reference strain. (DOC 42 KB) [file 12864_2014_6397_MOESM1_ESM.doc]

**Supplemental Table 1. Prediction of accessory elements in reference *P. aeruginosa* genomes using AGEnt and comparison to regions excluded from core genome generation in each reference strain.**

|  | AGEnt elements | | | | | | Comparison to non-core segments | | |
| --- | --- | --- | --- | --- | --- | --- | --- | --- | --- |
| Genome | Segments | Total Size (bp) | % of Genome | Average Size (bp) | Average G+C content (%) | CDS | # bases overlapping  (% of total AGE finder, % of total non-core) | # bases unique to AGEnt  (% of total AGEnt) | # bases unique to non-core  (% of total non-core) |
| 19BR | 190 | 899,788 | 13.34 | 4,736 | 60.9 | 767 | 898,649 (99.87, 99.26) | 1,139 (0.13) | 6,729 (0.74) |
| 213BR | 192 | 880,008 | 13.10 | 4,583 | 61.0 | 739 | 878,869 (99.87, 99.25) | 1,139 (0.13) | 6,668 (0.75) |
| B136-33 | 173 | 578,570 | 9.01 | 3,344 | 60.8 | 474 | 576,806 (99.70, 99.41) | 1,764 (0.30) | 3,445 (0.59) |
| DK2 | 168 | 637,884 | 9.96 | 3,797 | 60.5 | 590 | 636,893 (99.84, 99.18) | 991 (0.16) | 5,264 (0.82) |
| LESB58 | 190 | 768,880 | 11.65 | 4,047 | 61.6 | 667 | 767,635 (99.84, 99.39) | 1,245 (0.16) | 4,673 (0.61) |
| M18 | 184 | 503,582 | 7.96 | 2,737 | 61.6 | 380 | 502,070 (99.70, 98.60) | 1,512 (0.30) | 7,132 (1.40) |
| NCGM2.S1 | 195 | 914,697 | 13.52 | 4,691 | 60.9 | 750 | 912,333 (99.74, 99.33) | 2,364 (0.26) | 6,161 (0.67) |
| PA7 | 457 | 1,154,544 | 17.52 | 2,526 | 62.1 | 581 | 1,151,156 (99.71, 96.56) | 3,388 (0.29) | 40,956 (3.44) |
| PA14 | 179 | 696,220 | 10.65 | 3,889 | 60.6 | 1054 | 696,157 (99.99, 99.42) | 63 (0.01) | 4,061 (0.58) |
| PACS2 | 181 | 663,647 | 10.22 | 3,667 | 61.1 | 433 | 662,306 (99.80, 99.39) | 1,341 (0.20) | 4,042 (0.61) |
| PAO1 | 183 | 427,473 | 6.82 | 2,336 | 61.5 | 345 | 426,434 (99.76, 99.14) | 1,039 (0.24) | 3,686 (0.86) |
| RP73 | 177 | 517,233 | 8.16 | 2,922 | 61.3 | 455 | 516,341 (99.83, 99.74) | 892 (0.17) | 1,351 (0.26) |
